# An in vitro comparison of PDGF‐BB release from composite graft materials containing leukocyte‐rich platelet‐rich fibrin and platelet‐derived growth factor‐BB and β‐tricalcium phosphate

**DOI:** 10.1002/jper.70157

**Published:** 2026-07-02

**Authors:** Farahnaz Fahimipour, Maggie A. Misch‐Haring, Ramzi V. Abou Arraj, Nicolaas C. Geurs, Maria L. Geisinger

**Affiliations:** ^1^ Department of Periodontology University of Alabama at Birmingham Birmingham Alabama USA; ^2^ Private Practice, Limited to Periodontics Sarasota Florida USA; ^3^ University of Alabama at Birmingham Birmingham Alabama USA

**Keywords:** growth factors, L‐PRF membrane, PDGF‐BB, β‐TCP

## Abstract

**Background:**

The clinical efficacy of growth factors in regenerative procedures may depend on both dosage and sustained delivery during critical phases of healing. This study aimed to evaluate in vitro release kinetics of platelet‐derived growth factor‐BB (PDGF‐BB) from various graft materials, including recombinant human PDGF‐BB (rhPDGF‐BB) combined with beta‐tricalcium phosphate (β‐TCP), leukocyte platelet‐rich fibrin (L‐PRF) with β‐TCP (PRF bone block), L‐PRF bone block with added rhPDGF‐BB, and L‐PRF membranes alone.

**Methods:**

Whole blood samples were collected from five healthy adult donors. L‐PRF membranes and autologous liquid fibrinogen were prepared according to manufacturer's protocols. Five samples of each of the following were prepared: L‐PRF membranes, L‐PRF + β‐TCP with or without rhPDGF‐BB, and β‐TCP + rhPDGF‐BB. All materials were incubated at 37°C, and 2 mL aliquots of supernatant were collected at 1 hour, 8 hours, 1 day, 3 days, and 10 days. PDGF‐BB concentrations were quantified using enzyme‐linked immunosorbent assay (ELISA), and data were analyzed using a linear mixed effects model with Tukey's post hoc test.

**Results:**

Significantly higher PDGF‐BB concentrations were observed in samples containing rhPDGF‐BB at all‐time points. Further, the L‐PRF + rhPDGF‐BB + β‐TCP group demonstrated a gradual and sustained release from 1 hour to 10 days. In contrast, the rhPDGF‐BB + β‐TCP group showed an early peak followed by minimal release after day 3.

**Conclusion:**

The incorporation of L‐PRF into β‐TCP scaffolds containing rhPDGF‐BB resulted in prolonged and more controlled PDGF‐BB release. Composite graft formulation may maintain growth factor availability during early and intermediate phases of healing.

**Plain language summary:**

This study was designed to determine how different materials release a healing protein, PDGF‐BB, over time. This protein helps tissues regenerate, and its effectiveness depends on both how much is delivered and how long it stays active during healing. Four combinations of composite graft materials were tested using blood from healthy donors: (1) laboratory‐produced version PDGF‐BB with a synthetic hard tissue graft (β‐TCP), (2) autologous blood product (L‐PRF) with β‐TCP, (3) L‐PRF with β‐TCP plus added laboratory‐produced PDGF‐BB, and (4) L‐PRF alone.

PDGF‐BB release was measured at different times over 10 days. Composite grafts with added laboratory‐produced PDGF‐BB released more of the protein overall. The combination of L‐PRF, β‐TCP, and synthetic PDGF‐BB released the protein slowly and steadily over time. The synthetic PDGF‐BB with β‐TCP alone released a lot early on, but very little after day 3.

Based upon these findings and within the limitation of this study, use of composite grafts containing L‐PRF with β‐TCP and laboratory‐produced PDGF‐BB may help improve healing by keeping the growth factor available longer during the most important stages of tissue repair. Further clinical studies are necessary to determine the comparative clinical benefits of such composite grafts.

## 1 INTRODUCTION

Successful wound healing following injury or surgical therapy requires the orchestrated interplay of cells, signaling molecules, and extracellular matrix components. Among these, growth factors play a critical role in directing the biological processes of inflammation, angiogenesis, and tissue regeneration.^1^ One of the most well‐characterized growth factors in regenerative dentistry is platelet‐derived growth factor‐BB (PDGF‐BB), which is known for its potent chemotactic and mitogenic effects on mesenchymal progenitor cells, including osteoblasts and fibroblasts.^2^ PDGF‐BB not only promotes cellular migration and proliferation but also supports angiogenesis by upregulating vascular endothelial growth factor (VEGF) and integrin expression—making it particularly valuable in applications that aim to regenerate alveolar bone and soft tissues.^3^, ^4^

Advances in recombinant protein technology have enabled the clinical application of PDGF‐BB as a therapeutic biologic during regenerative therapy. Recombinant human PDGF‐BB (rhPDGF‐BB) is one of only two growth factors currently approved by the U.S. Food and Drug Administration (FDA) for oral regenerative procedures, alongside recombinant human bone morphogenetic protein‐2 (rhBMP‐2).^1^ rhPDGF‐BB is commercially available for use in oral regeneration in combination with beta‐tricalcium phosphate (β‐TCP), a synthetic, biocompatible, and osteoconductive scaffold (GEM 21S, Geistlich Pharma).^2^, ^5^, ^6^ This formulation has demonstrated clinical efficacy in enhancing both soft and hard tissue healing in a range of oral surgical applications, including guided tissue regeneration (GTR), guided bone regeneration (GBR), sinus floor elevation, lateral ridge augmentation, and alveolar ridge preservation.^7^, ^8^, ^9^, ^10^ Furthermore, the durability of these outcomes has been substantiated in long‐term investigations. In a 36‐month randomized, controlled, double‐masked clinical trial, Nevins et al. reported that rhPDGF‐BB treatment resulted in a higher percentage of sites achieving clinical attachment level (CAL) gain > 2.7 mm and radiographic bone fill > 1.5 mm compared to control therapy, demonstrating its potential for significant long‐term improvements in regenerative potential.

To increase stability of rhPDGF‐BB during the initial healing period, researchers have investigated various biomaterial carriers that can modulate and extend the bioavailability of rhPDGF‐BB. β‐TCP, while providing a volumetrically stable and osteoconductive scaffold, has demonstrated limitations in retaining growth factors over time, often leading to a rapid initial release without sustained delivery. To improve growth factor retention and enhance the biological activity of graft materials, autologous platelet‐rich fibrin (PRF) has emerged as a promising adjunct.^11^, ^12^

PRF is a second‐generation, autologous blood product (ABP) that is rich in platelets, leukocytes, and embedded within a fibrin matrix. Unlike platelet‐rich plasma (PRP), PRF does not require anticoagulants or exogenous biochemical additives, and its natural polymerized structure allows for the gradual release of multiple endogenous growth factors, including PDGF‐BB, TGF‐β1, VEGF, and EGF.^13^ These molecules support angiogenesis, soft tissue healing, and early bone formation. Of particular interest is leukocyte‐rich PRF (L‐PRF), which has demonstrated sustained growth factor release over several days and has been widely used in both periodontal and implant site development procedures.^11^, ^12^, ^13^, ^14^, ^15^

Recent innovations have led to the widespread clinical use of L‐PRF bone blocks (or “sticky bone”), which combine particulate bone graft substitutes such as beta‐tricalcium phosphate (β‐TCP) with chopped L‐PRF membranes and autologous liquid fibrinogen. These composite grafts are often reported to increase ease of clinical use through improved graft cohesiveness and moldability, offering enhanced graft stability and space maintenance within defects. However, L‐PRF alone lacks osteoinductive properties and typically resorbs within two to three weeks, limiting its utility as a stand‐alone regenerative material for larger or more complex defects. Therefore, L‐PRF is often combined with a mineralized scaffold to provide both biological activity and structural integrity.^14^

Despite the demonstrated benefits of rhPDGF‐BB, one of the key limitations of rhPDGF‐BB therapy lies in its release profile. When delivered without a suitable carrier system, rhPDGF‐BB may undergo rapid clearance from the surgical site, resulting in a short‐lived burst of growth factor activity that may not support the extended timeline required for complete tissue regeneration. As such, the method of delivery, including both the dosage and duration of growth factor release, is a critical determinant of clinical success. Sustained, localized release of PDGF‐BB is believed to better mimic the physiological healing environment and may yield improved regenerative outcomes.^16^, ^17^

In this context, L‐PRF has been proposed as a promising adjunctive biomaterial to extend and modulate the local availability of growth factors, including PDGF. L‐PRF is an autologous, fibrin‐rich matrix that can gradually release endogenous bioactive molecules over time, potentially complementing the regenerative effects of rhPDGF‐BB. Recent systematic reviews and network meta‐analyses by the American Academy of Periodontology (AAP) have highlighted the efficacy of biologics, including PRF, across a variety of clinical indications such as periodontal intrabony defects, root coverage, gingival augmentation, and alveolar ridge preservation.^7^, ^18^, ^19^ Collectively, these findings underscore the potential of L‐PRF to serve as a biologically active scaffold that not only supports wound healing but also enhances the performance of recombinant biologics through sustained, synergistic delivery.

An emerging strategy involves combining L‐PRF with exogenous rhPDGF‐BB and *β*‐TCP, potentially creating a dual‐delivery system that merges the sustained release capacity of PRF with the robust regenerative stimulus of rhPDGF‐BB and the osteoconductivity of β‐TCP. Such a formulation could simultaneously address multiple challenges in regenerative therapy: (1) ensuring an effective and sustained growth factor release, (2) improving handling properties and graft stability during surgery, and (3) enhancing the overall healing response in alveolar bone defects.^20^, ^21^

While these concepts have been applied in clinical practice, there is limited empirical data comparing the release kinetics of PDGF‐BB from L‐PRF alone, β‐TCP alone, or composite regenerative materials with or without exogenous rhPDGF‐BB. Understanding release profiles is essential for optimizing the impact of adjunctive growth factors on wound healing and regenerative outcomes. These assessments will allow clinicians to consider the composition of bone graft materials to improve outcomes and clinical decision‐making for regenerative procedures.^12^

This study sought to investigate in vitro interval release kinetics of PDGF‐BB from various bone graft constructs, including L‐PRF alone, L‐PRF + β‐TCP, rhPDGF‐BB + β‐TCP, and L‐PRF + rhPDGF‐BB + β‐TCP. In addition to growth factor release, a qualitative assessment of handling properties was conducted to determine whether the addition of rhPDGF‐BB altered the physical characteristics of the L‐PRF bone block. This investigation seeks to inform the design of biologically enhanced graft materials that offer both favorable release profiles and practical handling characteristics, with the goal of improving regenerative outcomes in clinical oral surgery.

## 2 MATERIALS AND METHODS

### 2.1 Whole blood collection

This study protocol was approved by the institutional review board (IRB) at the University of Alabama at Birmingham (UAB) (IRB approval number IRB‐300005971) and registered on ClinicalTrials.gov (NCT04964466). All participants were enrolled following the provision of written informed consent. This study was conducted in accordance with the Helsinki Declaration. Whole blood was collected from a total of five healthy adult volunteers using standard venipuncture techniques. Eligible individuals were English‐speaking adults capable of understanding and signing the informed consent document. Exclusion criteria included the presence of systemic disease, current use of medications, tobacco use, or any physical, anatomical, or psychological condition that could contraindicate oral surgical procedures or negatively impact wound healing (Table 1). All included patients were systemically healthy patients (American Society of Anesthesia perioperative risk category 1).

**TABLE 1:** Inclusion and exclusion criteria.

Inclusion criteria	Exclusion criteria
English speaking	Non‐English speaking
Able to read and understand informed consent document	Smokers/tobacco users
Systemically healthy, non‐smoker, no medications	Patients with systemic pathologies, anatomical considerations, or conditions contraindicating oral surgical procedures or adversely affecting wound healing

For each participant, six vacutainer tubes (BD, Franklin Lakes, NJ) were collected: three 9 mL red‐top tubes containing a clot activator and three 9 mL white‐top tubes with no additives. The collected blood was used to prepare L‐PRF, autologous liquid fibrinogen, and composite “sticky bone” graft constructs for subsequent experimental analysis. Each participant contributed to one sample per experimental group.

### 2.2 Preparation of L‐PRF membranes

One red‐top and one white‐top tube from each donor were processed using the IntraSpin centrifugation system (BioHorizons Inc., Birmingham, AL) at 2700 rpm. The white‐top tube was removed after 3 minutes to extract the liquid fibrinogen, while the red‐top tube continued centrifugation for a total of 12 minutes. The resulting L‐PRF clot was carefully separated from the red blood cell layer and compressed using the Xpression Box (BioHorizons Inc.) to form a membrane. The membrane was then mixed with 1 mL of autologous liquid fibrinogen using sterile technique and transferred to a 12‐well tissue culture plate (Corning Inc., Kennebunk, ME).

### 2.3 Preparation of PRF + β‐TCP (“sticky bone”)

For the L‐PRF + β‐TCP group, one red‐top and one white‐top tube of blood per donor were processed as described above. The L‐PRF membrane was cut into approximately 5 mm^2^ pieces and combined with 0.5 cc of β‐TCP (GUIDOR easy‐graft CLASSIC, Sunstar). A total of 1 mL of liquid fibrinogen was aspirated using a sterile 18G 1.5‐inch needle and mixed with the PRF and β‐TCP to create a cohesive “sticky bone” graft. The resulting constructs were placed into individual wells of a 12‐well culture plate.

### 2.4 Preparation of rhPDGF‐BB + β‐TCP

To prepare the rhPDGF‐BB + β‐TCP group, 0.15 µg (0.5 mL) of rhPDGF‐BB (GEM 21S, Luitpold Pharmaceuticals) was combined with 0.5 cc of β‐TCP and allowed to hydrate in a sterile dish for 15 minutes. The hydrated graft complex was then transferred to a 12‐well culture plate.

### 2.5 Preparation of L‐PRF + rhPDGF‐BB + β‐TCP composite

Two tubes (one red‐top and one white‐top) from each donor were processed to obtain PRF membranes and liquid fibrinogen. In parallel, 0.5 cc of rhPDGF‐BB was added to 0.5 cc of β‐TCP and allowed to hydrate for 15 minutes. The L‐PRF membrane was sectioned and mixed with the rhPDGF‐BB‐saturated β‐TCP; 1 mL of liquid fibrinogen was added to the mixture using a sterile syringe, forming a cohesive composite graft. These constructs were then placed in 12‐well culture plates for incubation.

All prepared graft materials were photographed and qualitatively assessed by a single examiner (M.M.) for a subjective assessment of clinical handling characteristics. Each preparation was assigned a positive, neutral, or negative value for cohesiveness, moldability, and ease of manipulation under simulated surgical conditions. Specifically, composite graft preparations were moved from one medicine cup to another and packed using surgical instrumentation employed for ridge preservation procedures (Figure 1).

**FIGURE 1:**
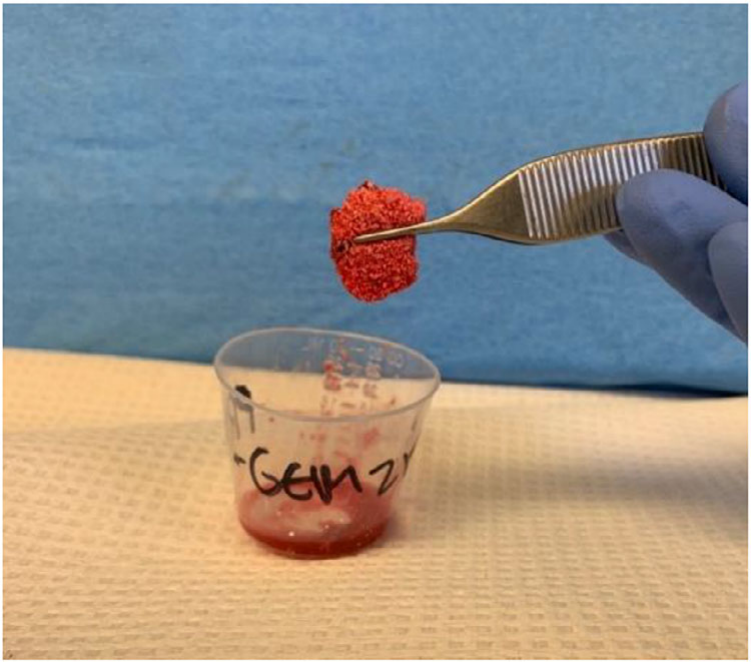
Example photograph of examiner (M.M.) performing quantitative assessment of clinical handling properties for composite graft materials.

### 2.6 Growth factor release and ELISA quantification

Each graft sample was incubated in 2 mL of phosphate‐buffered saline (PBS) at 37°C to simulate physiological conditions. At predetermined intervals (1 hour, 8 hours, 1 day, 3 days, and 10 days), 2 mL of the supernatant was collected and replaced with fresh PBS. All collected samples were immediately frozen at −80°C for batch analysis of pooled samples.

Quantification of PDGF‐BB in the collected supernatants to assess interval growth factor release was performed using a commercially available enzyme‐linked immunosorbent assay (ELISA) kit (Quantikine Human PDGF‐BB; R&D Systems, Minneapolis, MN; Cat# DBB00), following the manufacturer's instructions. All measurements were conducted in duplicate, and the average concentration was recorded for each sample.

### 2.7 Statistical analysis

A linear mixed effects model was employed to evaluate differences in PDGF‐BB release across groups and time points. Post hoc comparisons were performed using Tukey's test to account for multiple comparisons. Statistical significance was defined as *p* < 0.05. All analyses were conducted using appropriate statistical software packages (SPSS, IBM Corp.; or R, R Foundation for Statistical Computing).

## 3 RESULTS

Whole blood samples were collected from five systemically healthy volunteers. The study participants were three males and two females ranging in age from 52 to 70 years old. Participants all identified as Caucasian/White. While no differences were noted in findings based upon patient demographics, including age and biological sex, the number of individuals who provided samples was small and the group was homogeneous. This study was not powered to determine statistical differences based upon demographics. Detailed results of the between‐group comparisons are presented in Table S1 in the online *Journal of Periodontology*. Quantitative analysis of PDGF‐BB concentrations over a 10‐day period revealed no statistically significant differences between the L‐PRF‐alone and L‐PRF + β‐TCP groups at any of the measured time points (1 hour, 8 hours, 1 day, 3 days, and 10 days) (Figure 2). Both groups demonstrated a similar interval growth factor release profile, characterized by an initial moderate release followed by a gradual decline in growth factor availability at an 8‐hour time point and relatively stable release to 10 days. These findings indicate that the addition of β‐TCP to L‐PRF does not significantly alter the release kinetics of endogenously derived PDGF‐BB. It was also noted that PDGF‐BB release was significantly lower at all‐time points for composite grafts that did not contain exogenous PDGF than those composed of L‐PRF only.

**FIGURE 2:**
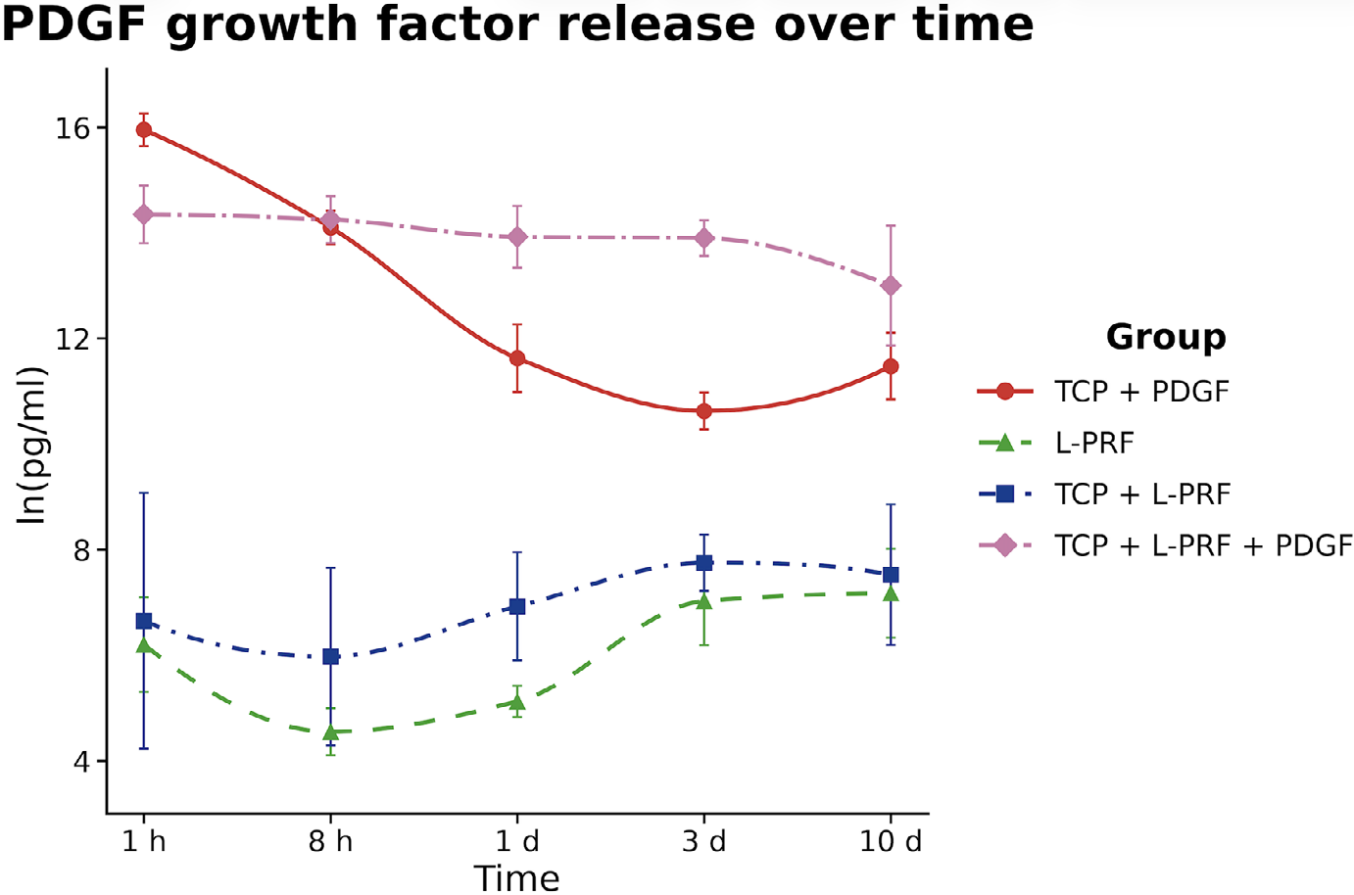
PDGF‐BB release kinetics over time among different biomaterial formulations. The graph shows the log‐transformed concentration of PDGF‐BB (log(pg/mL)) released from four groups: TCP+PDGF (red solid line), PRF (green dashed line), TCP+PRF (blue dashed dot line), and TCP+PRF+PDGF (purple long‐short dash line), measured at 1 hour, 8 hours, 1 day, 3 days, and 10 days. PDGF‐BB, platelet‐derived growth factor‐BB; PRF, platelet‐rich fibrin; TCP, tricalcium phosphate.

In contrast, groups supplemented with recombinant PDGF‐BB, namely, rhPDGF‐BB + β‐TCP and L‐PRF + rhPDGF‐BB + β‐TCP, exhibited significantly higher levels of PDGF‐BB at all evaluated time points (*p* < 0.05) when compared to the L‐PRF‐only and L‐PRF + β‐TCP groups (Figure 2). At the 1‐hour time point, the average concentration of PDGF‐BB in the L‐PRF‐alone group was approximately 662 pg/mL, whereas the rhPDGF‐BB + β‐TCP group reached a concentration of 9,071,593 pg/mL. Although L‐PRF‐based samples continued to release growth factors over time, the rhPDGF‐BB–supplemented groups consistently maintained significantly higher levels of PDGF‐BB throughout the entire observation period, confirming the robust and sustained release potential of exogenously applied rhPDGF‐BB.

When comparing the two rhPDGF‐BB–containing groups, distinct differences in release patterns were observed (Figure 2). No statistically significant differences were found between the L‐PRF + rhPDGF‐BB + β‐TCP and rhPDGF‐BB + β‐TCP groups at the early time points (1 hour and 8 hours). However, at 1 day and 3 days, the L‐PRF + rhPDGF‐BB + β‐TCP group demonstrated significantly higher levels of PDGF‐BB release (*p* < 0.05). This group also exhibited a more gradual and sustained release profile from 1 hour to 10 days, whereas the rhPDGF‐BB + β‐TCP group showed a pronounced early release between 8 hours and 1 day, followed by minimal additional release after day 3. By day 10, no significant differences were observed between the two groups. These findings suggest that the incorporation of L‐PRF into the rhPDGF‐BB + β‐TCP composite may enhance the modulation of growth factor delivery, allowing for more controlled and prolonged release.

Subjective qualitative assessment of graft handling revealed no discernible differences between the L‐PRF + β‐TCP and L‐PRF + rhPDGF‐BB + β‐TCP preparations. For all assessed qualitative measures (i.e. cohesiveness, moldability, and ease of manipulation), both L‐PRF + rhPDGF‐BB + β‐TCP and L‐PRF + β‐TCP were assessed positively with no differences noted between composite graft materials. Both graft types demonstrated excellent cohesion, moldability, and ease of manipulation, supporting their suitability for clinical application. These results should be interpreted with caution, however, given the limited number of samples and the subjective nature of the assessments. Within the limitations of this study, the addition of rhPDGF‐BB was not assessed to negatively impact the physical characteristics of the L‐PRF bone block preparation.

## 4 DISCUSSION

The aim of this study was to compare the interval release kinetics of PDGF‐BB from four different grafting formulations: L‐PRF alone, L‐PRF + β‐TCP, rhPDGF‐BB + β‐TCP, and L‐PRF + rhPDGF‐BB + β‐TCP. A qualitative evaluation was also conducted to assess whether the addition of rhPDGF‐BB to β‐TCP + L‐PRF altered the handling characteristics of the resulting “sticky bone” graft compared to β‐TCP + L‐PRF alone.

The findings of this investigation demonstrate that the addition of exogenous rhPDGF‐BB resulted in higher levels of PDGF‐BB release at all‐time points, including composite grafts containing L‐PRF, which has been shown to contain autologous PDGF‐BB. Although the L‐PRF‐containing groups exhibited a slower and more gradual release over time, the absolute concentration of PDGF‐BB remained markedly lower than that of rhPDGF‐BB–supplemented samples, even at the 10‐day mark.

L‐PRF has been employed clinically as an adjunct for oral regenerative procedures. As an autologous product with the capacity to deliver a variety of endogenous growth factors from platelet α‐granules, including PDGF‐BB, it has been shown to demonstrate enhanced hard and soft tissue regeneration when combined with other grafts. However, the results from this study reaffirm the potential limitations of L‐PRF alone in achieving therapeutically optimal concentrations of PDGF‐BB. In addition, due to the biological variability inherent in autologous preparations, L‐PRF may lack consistency and growth factor content can vary significantly between individuals.^22^ Prior research has also shown that while L‐PRF may enhance early wound healing and soft tissue maturation, its direct osteogenic or osteoinductive potential may be limited.^11^, ^17^

A more nuanced comparison between the rhPDGF‐BB + β‐TCP and L‐PRF + rhPDGF‐BB + β‐TCP groups revealed significant differences in PDGF‐BB release at days 1 and 3, but not at 1 hour, 8 hours, or day 10. Both groups exhibited an initial burst release at the 1‐hour mark; however, the addition of L‐PRF to the rhPDGF‐BB + β‐TCP preparation appeared to modulate the kinetics of release, resulting in a more gradual and sustained PDGF‐BB delivery over the first 3 days. This effect is likely attributable to the fibrin network in L‐PRF, which may serve as a temporary reservoir, retaining the growth factor within its matrix and releasing it progressively as the fibrin undergoes degradation and remodeling, akin to the physiological breakdown of a natural blood clot.^14^

Within the limitations of this study's subjective handling analysis, the incorporation of rhPDGF‐BB into the L‐PRF + β‐TCP “sticky bone” construct was not judged to compromise its handling characteristics. Both L‐PRF + β‐TCP and L‐PRF + rhPDGF‐BB + β‐TCP preparations were assessed to demonstrate excellent clinical workability, moldability, and cohesive properties. These qualities are critical for surgical ease, graft stability, and space maintenance within osseous defects. While the in vitro simulations used to assess handling characteristics were limited and likely do not reflect all clinical applications for L‐PRF blocks (“sticky bone”), the addition of rhPDGF‐BB with L‐PRF and β‐TCP did not substantially alter cohesiveness, moldability, and workability. Therefore, the use of L‐PRF bone blocks with rhPDGF‐BB and β‐TCP may offer dual benefits: enhanced growth factor delivery and superior physical graft characteristics and ease of use.

An effective carrier for recombinant growth factors must support both biologically optimal release and physiologic integration.^23^ Optimally, growth factor release during key healing periods would allow for activation of subsequent downstream healing cascades that enhance neovascularization and cell recruitment for enhanced healing. An ideal carrier would provide space maintenance, osteoconductivity, biocompatibility, the ability to bind and subsequently release at appropriate times growth factors, and a degradation profile aligned with new bone formation. β‐TCP has been shown to fulfill many of these criteria, and its use as a scaffold for rhPDGF‐BB is well documented. However, a considerable limitation of β‐TCP as a sole carrier is its tendency for rapid release and potential in vivo clearance of the growth factor, which may reduce its therapeutic efficacy over time.^24^, ^25^

The integration of L‐PRF to create “sticky bone” can offer several clinical advantages in addition to biological enhancement. Composite grafts containing L‐PRF and liquid fibrinogen are cohesive and pliable and, as such, simplify manipulation and placement, particularly in irregular defect morphologies. Additionally, L‐PRF contributes additional autologous growth factors such as TGF‐β, VEGF, EGF, and IGF in a time‐dependent manner similar to natural blood clot degradation that can complement the effects of exogenously added rhPDGF‐BB.^26^ The present findings suggest that combining L‐PRF with rhPDGF‐BB and β‐TCP may extend growth factor release over several days, potentially supporting a more physiologically appropriate healing trajectory. Although the clinical significance of this extended release must be confirmed in vivo, the results warrant further exploration of this combinatory approach for its potential to enhance both hard and soft tissue regenerative outcomes.^27^, ^28^

To date, limited studies have evaluated the combined use of L‐PRF, β‐TCP, and rhPDGF‐BB as a composite grafting material. While such materials are already in use clinically, the scientific literature lacks data on their synergistic effects in growth factor delivery and clinical efficacy. It is also unclear if such composite grafts are particularly advantageous in specific clinical scenarios, such as for patients with systemic disease impacting would healing or when graft sites are large. This study is, to our knowledge, the first to investigate PDGF‐BB release dynamics from these combined systems, offering foundational insight into their potential advantages.^21^, ^29^

One important limitation of this study is the variability observed in growth factor content across donors. While no statistically significant differences were noted in growth factor release based upon demographics in this study, the low number of individuals contributing whole blood in this investigation was likely under powered to see such differences. Thus, the inter‐individual variation may be influenced by factors such as age, biologic sex, and the time elapsed between venipuncture and centrifugation. Prior research has shown that L‐PRF membrane size and composition are sensitive to preparation protocols, with delays in centrifugation potentially reducing the separation efficiency and affecting fibrin architecture and cell entrapment. Such differences in membrane structure may significantly influence the timing and quantity of growth factor release.^21^ While standardized centrifugation with calibrated centrifuges and standardized preparation protocols was utilized in this study with a FDA approved preparation system, there are a variety of systems available commercially and other L‐PRF centrifugation and preparation protocols in common clinical use. Given these variations, the findings of this study may not be universally generalizable and are specific to the preparation protocol and donor characteristics used here.

Interpretation of these findings should further consider that this study was designed to examine the interval release between time points during the assay as the entire 2 mL supernatant was removed and replaced at each assessment time point. It is, therefore, impossible to determine within that period if the release occurred evenly throughout or in bursts. Additionally, because rhPDGF‐BB was exogenously added in some groups, differences between groups could relate to either carrier‐mediated release kinetics at the intervals assessed and/or initial loading conditions and the current experiment was not designed to determine such differences. Future research could elucidate these questions through assessment of loading characteristics and more continuous assessments of release kinetics.

Future research should include larger‐scale in vitro studies and in vivo validation using both animal models and clinical trials. Investigations should also explore the influence of patient‐specific variables and processing parameters on L‐PRF quality and performance. Additionally, determining the optimal clinical indications for combining L‐PRF with β‐TCP and rhPDGF‐BB will be critical, particularly given the added cost associated with recombinant biologics. Identifying defect types or patient profiles most likely to benefit from sustained PDGF‐BB release may allow for more targeted, cost‐effective applications in clinical practice.

In conclusion, this study provides novel evidence that L‐PRF can modulate the release profile of rhPDGF‐BB when used in combination with β‐TCP, resulting in a more sustained delivery of the growth factor over several days. While L‐PRF alone delivers limited amounts of PDGF‐BB, its incorporation into graft constructs may enhance both the biological and physical properties of the material. These findings support further investigation into the clinical potential of composite grafts containing L‐PRF, β‐TCP, and rhPDGF‐BB to improve regenerative outcomes in bone and periodontal therapies.

## 5 CONCLUSIONS

There is increasing interest in optimizing wound healing and enhancing hard tissue regeneration in complex alveolar reconstruction procedures. Recombinant human platelet‐derived growth factor‐BB (rhPDGF‐BB) is among the most extensively studied growth factors in periodontics and oral surgery. It is a potent stimulator of angiogenesis and significantly promotes the proliferation and migration of osteoblasts and periodontal cells—properties that are highly advantageous for achieving alveolar bone regeneration.

Within the limitations of this investigation, the findings of this study indicate that the release kinetics of PDGF‐BB can be favorably modulated through the incorporation of L‐PRF with β‐TCP as a delivery matrix. Specifically, combining L‐PRF with rhPDGF‐BB and β‐TCP results in a more sustained release profile of PDGF‐BB without compromising the desirable clinical handling properties of an L‐PRF bone block (“sticky bone”).

Future clinical investigations are necessary to determine clinical benefits of such composite grafts, but these findings indicate that the use of a composite graft containing L‐PRF, rhPDGF‐BB, and β‐TCP may allow for more prolonged and sustained PDGF‐BB release and enhance clinical outcomes in regenerative dentistry.

## AUTHOR CONTRIBUTIONS

Maria L. Geisinger and Maggie A. Misch‐Haring contributed to the inception of the study and analyzed the data. Maria L. Geisinger, Maggie A. Misch‐Haring, and Farahnaz Fahimipour contributed to manuscript preparation. All the authors made critical changes and gave final approval of the manuscript and agreed to be accountable for all aspects of the work.

## CONFLICT OF INTEREST STATEMENT

The authors declare no conflicts of interest.

## Supporting information




Supplementary Information
