# Supplementary material for: An in vitro comparison of PDGF‐BB release from composite graft materials containing leukocyte‐rich platelet‐rich fibrin and platelet‐derived growth factor‐BB and β‐tricalcium phosphate
Source: J Periodontol. 2026 Jul 2;97(10):2173–81. doi: 10.1002/jper.70157 (PMC13743498; doi:10.1002/jper.70157)
Supplement: Supplementary file 1 [file JPER-97-2173-s001.docx]

Table 1(Suppl): Mean log measure Differences Between Group Comparisons (**Bold** indicates non-significant results)

| Group Comparison | Time Point | Mean | Standard Error (SE) | Degrees of Freedom (df) | T ratio | p-value |
| --- | --- | --- | --- | --- | --- | --- |
| TCP.PDGF – PRF | 1 hour | 9.79 | 0.59 | 52.01 | 16.63 | 0.00 |
| TCP.PDGF – PRF | 8 hour | 9.32 | 0.59 | 52.01 | 15.82 | 0.00 |
| TCP.PDGF – PRF | 1 day | 6.02 | 0.59 | 52.01 | 10.23 | 0.00 |
| TCP.PDGF – PRF | 3 day | 3.77 | 0.93 | 52.01 | 4.05 | 0.00 |
| TCP.PDGF – PRF | 10 day | 3.44 | 0.93 | 52.01 | 3.70 | 0.00 |
| TCP.PDGF - PRF.TCP | 1 hour | 9.12 | 0.59 | 52.01 | 15.49 | 0.00 |
| TCP.PDGF - PRF.TCP | 8 hour | 8.05 | 0.59 | 52.01 | 13.68 | 0.00 |
| TCP.PDGF - PRF.TCP | 1 day | 4.93 | 0.59 | 52.01 | 8.37 | 0.00 |
| TCP.PDGF - PRF.TCP | 3 day | 2.76 | 0.93 | 52.01 | 2.97 | 0.02 |
| TCP.PDGF - PRF.TCP | 10 day | 3.48 | 0.93 | 52.01 | 3.74 | 0.00 |
| TCP.PDGF - PRF.TCP.PDGF | 1 hour | 1.30 | 0.59 | 52.01 | 2.21 | **0.14** |
| TCP.PDGF - PRF.TCP.PDGF | 8 hour | -0.09 | 0.59 | 52.01 | -0.16 | **1.00** |
| TCP.PDGF - PRF.TCP.PDGF | 1 day | -2.43 | 0.59 | 52.01 | -4.12 | 0.00 |
| TCP.PDGF - PRF.TCP.PDGF | 3 day | -3.30 | 0.93 | 52.01 | -3.54 | 0.00 |
| TCP.PDGF - PRF.TCP.PDGF | 10 day | -2.07 | 0.93 | 52.01 | -2.22 | **0.13** |
| PRF - PRF.TCP | 1 hour | -0.67 | 0.59 | 52.01 | -1.14 | **0.67** |
| PRF - PRF.TCP | 8 hour | -1.27 | 0.59 | 52.01 | -2.15 | **0.15** |
| PRF - PRF.TCP. | 1 day | -1.09 | 0.59 | 52.01 | -1.85 | **0.26** |
| PRF - PRF.TCP. | 3 day | -1.00 | 0.93 | 52.01 | -1.08 | **0.70** |
| PRF - PRF.TCP. | 10 day | 0.04 | 0.93 | 52.01 | 0.05 | **1.00** |
| PRF - PRF.TCP.PDGF | 1 hour | -8.49 | 0.59 | 52.01 | -14.42 | 0.00 |
| PRF - PRF.TCP.PDGF | 8 hour | -9.41 | 0.59 | 52.01 | -15.98 | 0.00 |
| PRF - PRF.TCP.PDGF | 1 day | -8.45 | 0.59 | 52.01 | -14.35 | 0.00 |
| PRF - PRF.TCP.PDGF | 3 day | -7.07 | 0.93 | 52.01 | -7.59 | 0.00 |
| PRF - PRF.TCP.PDGF | 10 day | -5.51 | 0.93 | 52.01 | -5.92 | 0.00 |
| PRF.TCP - PRF.TCP.PDGF | 1 hour | -7.82 | 0.59 | 52.01 | -13.28 | 0.00 |
| PRF.TCP - PRF.TCP.PDGF | 8 hour | -8.15 | 0.59 | 52.01 | -13.83 | 0.00 |
| PRF.TCP - PRF.TCP.PDGF | 1 day | -7.36 | 0.59 | 52.01 | -12.50 | 0.00 |
| PRF.TCP - PRF.TCP.PDGF | 3 day | -6.06 | 0.93 | 52.01 | -6.51 | 0.00 |
| PRF.TCP - PRF.TCP.PDGF | 10 day | -5.55 | 0.93 | 52.01 | -5.96 | 0.00 |
